# Biopsychosocial and organizational factors explaining professional engagement

**DOI:** 10.3389/fpubh.2026.1906716

**Published:** 2026-08-07

**Authors:** Tania Gaspar, Fábio Botelho Guedes, Ana Cerqueira, Elisa Aires, Dulce Pinto, Natália Costa, Lúcia Gomes, Inês Jongenelen

**Affiliations:** 1 HEI-Lab – Digital Human-Environment Interaction Labs, SPIC – Psychology, Innovation and Knowledge Service, Lusófona University, Lisbon/Porto, Portugal; 2 Faculty of Medicine, Environmental Health Institute (ISAMB), University of Lisbon (FMUL), Lisbon, Portugal; 3 Portuguese Lab for Healthy Workplaces (LABPATS)/Aventura Social Association (ASA), Lisbon, Portugal; 4 ISLA Gaia – Polytechnic Institute of Management and Technology, Vila Nova de Gaia, Portugal; 5 REMIT – Research on Economics, Management and Information Technologies, Porto, Portugal; 6 Research Centre for Sport, Physical Education, Exercise and Health (CIDEFES), Faculty of Physical Education and Sport (FEFD), Lusófona University, Lisbon, Portugal; 7 Research Centre for Sport Research, Training, Innovation and Intervention (CIFI2D), Oporto University, Porto, Portugal; 8 Center for Psychology at University of Porto, Faculty of Psychology and Educational Sciences, University of Porto, Porto, Portugal

**Keywords:** generations, health promotion, healthy work environments, mental health, professional engagement, psychosocial risks

## Abstract

**Introduction:**

Work environments can expose professionals to psychosocial risks affecting mental health, well-being, and engagement. Engagement is key to organizational performance, job satisfaction, and talent retention, and is shaped by individual, organizational, and contextual factors.

**Method:**

The present study is embedded in the Laboratory of Healthy Work Environments (LABPATS) and aims to analyze the relationship between professional engagement, sociodemographic and organizational factors, and personal and lifestyle factors, while considering generational specificities. 8,348 professionals participated in this study, the majority of whom were female (65.1%), aged 18–78 years (*M* = 44.43; *SD* = 10.80).

**Results:**

Age, satisfaction with compensation, organizational ethics and values, a healthy psychosocial environment, community engagement, positive health behaviors, and stress management were associated with higher professional engagement. Psychosocial risks and teleworking showed negative associations, varying across generations. Baby Boomers and Generation X emphasize physical conditions, job stability and organizational climate, while Generations Y and Z are more sensitive to psychosocial environments and relationships. Older cohorts prioritize tangible aspects and security, whereas younger generations emphasize workplace relationships and the psychological climate.

**Discussion:**

Organizations should prioritize healthy and ethical work environments that support mental health and well-being. These findings guide decision-makers and policymakers in developing generation-sensitive interventions that improve professional engagement and sustainability.

## Introduction

Mental health in the workplace has gained increasing attention in scientific and social discussions. Research indicates that anxiety and depressive disorders are increasing among employees and are frequently associated with organizational changes that impact working conditions and job demands (1–4). High levels of physical and psychological distress among workers are associated with organizational factors, including low wages, long working hours, and job insecurity. They are also influenced by relational factors, such as inadequate social support, and leadership, limited decision-making autonomy, high demands, and workplace abuse (1–3, 5). In Europe, this issue is increasingly recognized as a public health concern because of its substantial individual, economic, and societal consequences. These include physical and mental health problems among workers, increased absenteeism, employee turnover, and higher costs related to work disability and pensions, as well as broader impacts on mortality and poverty (2, 4, 6–8). Current literature highlights that the psychosocial work environment is fundamental to maintaining professionals' mental health. At the same time, e growing evidence indicates that continuous exposure to unfavorable working conditions increases psychosocial risks and adversely affects professionals' health (2, 4, 8–10). Consequently, recent studies have shown that high work demands and intensity, pressure, and task accumulation negatively affect workers' wellbeing and performance, as well as organizational outcomes (2, 4). These factors contribute to the high levels of absenteeism observed in Europe.

It is estimated that 50% to 60% of work absence days are attributable to psychosocial risks with occupational stress affecting approximately one in four workers (8, 9). Occupational stress develops through prolonged exposure to adverse psychosocial conditions in the workplace. These risks include interpersonal conflicts, poor relationships with supervisors, and insufficient organizational support. Such conditions cause loss of control, job insecurity, and job dissatisfaction (6, 8, 10). Stress can also compromise workers' ability to perform their tasks safely (11). Assander et al. (6) reports that negative organizational cultures, high demands, and poor leadership are associated with greater job strain. In contrast, balanced workplaces protect workers, boost job satisfaction, and reduce health risks.

Research highlights the role of organizations in promoting healthy lifestyles to help manage occupational stress. Higher stress leads to risky health behaviors, such as unhealthy eating, alcohol or tobacco use, and poor sleep. These behaviors are considered poor coping strategies (11, 12). For example, Smirthdorf et al. (12) found that administrative workers at a university reported low levels of physical activity, poor sleep habits, and regular alcohol and tobacco consumption, behaviors that were closely associated with stress and job dissatisfaction. Conversely, healthy lifestyle habits, including adequate sleep, a balanced diet, and regular physical activity have been shown to protect against mental health problems and promote more effective coping strategies (13, 14).

A recent study by Jackson et al. (14) examined how workplace factors influence daily health behaviors. Barriers, such as limited access to healthy food, were linked to higher stress levels, whereas facilitators, including time and space for physical activity, promoted healthier behaviors and lower stress. The authors concluded that reducing workplace barriers and strengthening health-promoting resources can improve employee performance while reducing stress.

Favorable psychosocial work environments correlate with better worker health and job satisfaction. Resources such as development opportunities, recognition, fair pay, effective management, supportive leadership, autonomy, and social support can mitigate work-related health risks (6, 15). Individual factors such as self-efficacy and stress management skills also affect psychological wellbeing (8). Studies applying the Job Demands-Resources (JD-R) model to university have shown that factors such autonomy, quality leadership, and social support help promote wellbeing, job satisfaction, and work engagement (10, 16).

Work engagement and work–life balance are both vital for occupational wellbeing and stress reduction (7, 8, 10, 15, 16). Work engagement is a positive, fulfilling state associated with higher productivity, better organizational performance, and lower absenteeism and sick leave (10, 17, 18). This concept is generally defined as a positive and fulfilling work-related psychological state characterized by three core dimensions: vigor, dedication, and absorption. Vigor reflects high levels of energy, resilience, and persistence in facing work demands; dedication refers to a strong sense of involvement, enthusiasm, significance, and pride in one's work; and absorption describes a state of full concentration and immersion in work activities (19, 20). Work engagement is also strongly related to better physical and mental health, less depression and anxiety, and improved sleep (18).

Age diversity among workers affects resilience, interactions, job satisfaction, and productivity (21). Each generation has traits that shape its attitudes, relationships, values, and communication (21, 22). For example, Chakradhar et al. (21) found that professionals across three generations (Baby Boomers, Generation X, and Generation Y) reported high stress due to long working hours and competition. However, the sources of stress differ across generations. Baby Boomers tend to struggle with administrative demands, Generation X with balancing work and personal life, Generation Y with technology-related pressure, competition, and heavy workloads, and Generation Z, as the newest cohort in the workforce, faces its own distinct challenges. They are technology proficient, open to new approaches to learning, and focus on results. They also value the psychosocial environment, especially good relationships and a positive social climate (23–25).

Ignoring generational differences and failing to implement inclusive strategies may result in leadership practices that favor one generation over others. This may lead to unfairness, less work engagement, and lower job satisfaction (24). Still, there is limited research on how psychosocial risks, lifestyle, and work engagement relate across generations.

Conceptually, this study is grounded in an ecological perspective (26), integrating individual, organizational, and contextual determinants of professional engagement. The ecological model of human development conceptualizes development as the result of dynamic interactions between the person, proximal processes, context, and time (56, 57). From this perspective, professional engagement cannot be understood solely as the product of individual characteristics but rather as the outcome of interactions between workers and the contexts in which they are embedded (27, 55, 59, 60).

In line with resource-based approaches to work engagement, we examine how organizational resources (e.g., positive perceptions of ethics and values, a healthier psychosocial work environment, supportive community involvement) and individual resources (e.g., healthier behaviors and stress management capacity) are associated with higher engagement, while exposure to psychosocial risks and less favorable work arrangements (e.g., telework conditions) may be associated with lower engagement. Finally, given that different generational cohorts may hold distinct expectations and values regarding work, we explore whether the pattern of associations differs across Baby Boomers, Generation X, Generation Y, and Generation Z.

As previously noted, work engagement is widely defined as a positive work-related state characterized by vigor, dedication, and absorption (19, 20). In the present study, the construct of professional engagement is used to operationalize this positive and fulfilling relationship with work within an organizational context. Professional engagement is understood as a positive work-related state reflecting professionals' perceived sense of belonging, participation, and integration within their organization (27, 28). This focus on organizational integration and relational aspects of work is particularly suited to large-scale ecological studies examining psychosocial work environments, health, and wellbeing (9, 29). Thus, Thus, drawing on an ecological perspective, which emphasizes the dynamic interaction between individual characteristics and contextual factors, this study aims to examine links between professional engagement, organizational and individual factors, and to see how they connect to psychosocial risks and lifestyle. It also seeks to identify similarities and differences between generations now in the labor market.

## Method

### Participants

A total of 8,348 professionals were included in this study, of whom 65.1% (*N* = 5,462) were female, with ages ranging from 18 to 78 years (*M* = 44.43; *SD* = 10.80). The sample comprised professionals from several generations: 7.7% from the Baby Boomer generation (1946–1964), 48.9% from Generation X (1965–1980), 35.4% from Generation Y (1981–1996), and 8.0% from Generation Z (1997–2012) (30). The majority of respondents (65.4%) reported being dissatisfied with their remuneration.

## Variables and measures

Considering the objectives of the present study, the variables described in Table 1 were considered.

**Table 1:** Variables and measures under study.

Variables	Measures
Age	Continuous variable, measured in years, representing the participant's age.
Gender	1—Female; 2—Male
Satisfaction with compensation^a^	This is a 5-point scale adapted from Cantril (58), where 1 indicates the greatest dissatisfaction with compensation, and 5 indicates the greatest satisfaction.
Ethics and values^b^	Scale with 3 items, on a 5-point Likert scale, where 1 strongly disagrees, and 5 strongly agrees. Higher values indicate a lower risk associated with the professional entity's values and forms of relationship, reflecting on the safety and health of professionals (α = 0.90).
Professional engagement^b^	A 3-item scale using a 5-point Likert scale, where 1 indicates strong disagreement, and 5 indicates strong agreement. Higher values indicate a lower risk associated with professionals' sense of belonging, participation, and integration within their professional organization (α = 0.88).
Psychosocial environment^b^	A 6-item scale using a 5-point Likert scale, where 1 indicates strong disagreement, and 5 indicates strong agreement. Higher values indicate a lower risk associated with the psychosocial environment related to work content and leadership (α = 0.92).
Psychosocial risks related to mental health^b^	A 5-item scale using a 5-point Likert scale, where 1 indicates strong disagreement, and 5 indicates strong agreement. Higher values indicate a higher risk associated with work-related psychosocial factors affecting wellbeing and mental health (α = 0.85).
Physical environment^b^	A 3-item scale using a 5-point Likert scale, where 1 indicates strong disagreement, and 5 indicates strong agreement. Higher values indicate a lower risk associated with aspects of the physical work environment (α = 0.90).
Teleworking^b^	A 3-item scale using a 5-point Likert scale, where 1 indicates strong disagreement, and 5 indicates strong agreement. Higher values indicate a lower risk associated with the conditions and resources available for teleworking (α = 0.84).
Community involvement^b^	A 3-item scale using a 7-point Likert scale, where 1 indicates strong disagreement, and 7 indicates strong agreement. Higher values indicate a lower risk associated with engagement with the community (α = 0.82).
Risk behaviors^b^	Risk behaviors were assessed using questions about smoking habits, alcohol and other stimulant consumption, and screen time. Higher scores indicate greater engagement in risk behaviors (α = 0.70).
Health behaviors^a^	Health behaviors were assessed through questions on dietary habits (hydration, vegetable intake, soup consumption, fruit consumption), stress levels, sleep habits, and exercise practices. Higher values indicate healthier behaviors (α = 0.71).
Stress management^c^	A 4-item scale using a 5-point Likert scale assessed stress management capacity, with 1 indicating never/almost never and 5 indicating always/almost always. Higher scores on this scale correspond to greater stress management capacity (α = 0.82).

^a^ LABPATS Protocol, Gaspar et al. (53).

^b^ Healthy Work Environment Ecosystems Instrument (EATS), Gaspar et al. (28).

^c^ Cohen et al. (54).

## Procedures

This study is part of the Portuguese Laboratory of Healthy Work Environments (LABPATS) project. LABPATS examines in depth the health and wellbeing of professionals and organizations. The project aims to promote health and prevent occupational risks in Portugal, while maintaining a focus on performance (27).

Data collection was conducted between 2023 and 2024 and involved a representative sample of professionals from various organizations and professional bodies. The Ethics and Deontology Committee for Scientific Research (Cedic) of the School of Psychology and Life Sciences at Lusófona University of Humanities and Technologies approved the study (December 14, 2022). All participants volunteered after providing informed consent.

Questionnaire responses were collected online and anonymously. Data confidentiality and anonymity were ensured in accordance with data protection standards. Further details on the study's data collection procedures can be found in the final report by Gaspar et al. (27).

## Data analysis

Data were analyzed using the Statistical Package for the Social Sciences (SPSS), version 28 for iOS. To examine the relationships among the variables included in this study, Pearson's correlation coefficients were calculated., Multiple linear regressions were conducted to examine the relationship between professional engagement and sociodemographic, organizational, and personal/lifestyle factors. Additionally, generational differences were explored through the individual analysis of the four generations under study. Given the exploratory and explanatory aims of the study, hierarchical multiple regression was selected to identify the relative and incremental contribution of different groups of predictors to professional engagement. Predictors were entered in conceptually defined blocks, allowing the analysis to distinguish the contribution of sociodemographic characteristics, organizational factors, and personal/lifestyle variables. In this way, sociodemographic variables were entered first as distal background factors, followed by organizational variables representing work context resources and demands, and finally personal and lifestyle variables reflecting individual health-related resources. This hierarchical approach allows for estimating the incremental contribution of organizational and personal factors beyond basic sociodemographic characteristics. This strategy was considered appropriate for the present stage of analysis, while more complex latent models may be developed in future studies. A significance level of *p* < 0.05 was established.

## Results

The analysis of the correlations among the study variables (Table 2) revealed statistically significant associations between all variables under investigation. Specifically, work engagement was positively associated with organizational ethics and values, the psychosocial work environment, the physical work environment, community involvement, health behaviors, and stress management. In contrast, work engagement was negatively associated with psychosocial risks related to mental health.

**Table 2:** Descriptive statistics and correlations between study variables.

Variables	1	2	3	4	5	6	7	8	9	10
1. Ethics and values	–									
2. Professional engagement	0.52^***^	–								
3. Psychosocial environment	0.71^***^	0.64^***^	–							
4. Psychosocial risks related to mental health	−0.33^***^	−0.35^***^	−0.39^***^	–						
5. Physical environment	0.41^***^	0.29^***^	0.40^***^	−0.21^***^	–					
6. Teleworking	0.17^***^	0.12^***^	0.20^***^	−0.04^**^	0.21^***^	–				
7. Community involvement	0.55^***^	0.40^***^	0.53^***^	−0.31^***^	0.48^***^	0.22^***^	–			
8. Health behaviors	0.22^***^	0.22^***^	0.24^***^	−0.38^***^	0.19^***^	0.08^***^	0.25^***^	–		
9. Risk behaviors	−0.04^***^	−0.04^***^	−0.05^***^	0.10^***^	−0.05^***^	0.02^*^	−0.05^***^	−0.17^***^	–	
10. Stress management	0.17^***^	0.25^***^	0.22^***^	−0.30^***^	0.12^***^	0.05^***^	0.14^***^	0.32^***^	−0.10^***^	–

^***^p < 0.001; ^**^p < 0.01; ^*^p < 0.05.

In addition, organizational ethics and values, the psychosocial work environment, and community involvement were positively correlated with one another, whereas psychosocial risks related to mental health were negatively correlated with most of the organizational and individual variables examined. The strongest correlations were observed between organizational ethics and values and the psychosocial work environment (*r* = 0.71), between the psychosocial work environment and work engagement (*r* = 0.64), between organizational ethics and values and community involvement (*r* = 0.55), and between the psychosocial work environment and community involvement (*r* = 0.53). These findings underscore the importance of organizational factors in promoting work engagement.

From the inferential analysis of Table 3, it is observed that Model 1, which includes professional engagement and sociodemographic factors, explains approximately 7% of the variance, *F*_(3, 3493)_ = 81.588; *p* ≤ 0.001. Being older and having greater satisfaction with compensation are positively associated with professional engagement, whereas male gender is negatively associated with engagement.

**Table 3:** Multiple linear regression model of variables predicting professional engagement.

Models	Variables	*B*	Standard error	Beta	*t*
Model 1—Sociodemographic factors	(Constant)	3.06	0.08		36.90^***^
	Age	0.01	0.00	0.13	7.83^***^
	Gender	−0.13	0.03	−0.06	−3.72^***^
	Satisfaction with compensation	0.45	0.03	0.22	13.42^***^
	*R*^2^ = 0.065; *F* = 81.588 (3/3,493), *p* < 0.001				
Model 2—Organizational factors	(Constant)	1.12	0.12		9.48^***^
	Age	0.01	0.00	0.13	10.13^***^
	Gender	−0.12	0.03	−0.06	−4.61^***^
	Satisfaction with compensation	0.03	0.03	0.01	1.03
	Ethics and values	0.10	0.02	0.11	6.23^***^
	Psychosocial environment	0.52	0.02	0.53	29.57^***^
	Psychosocial risks related to mental health	−0.05	0.02	−0.04	−2.98^**^
	Physical environment	0.02	0.01	0.02	1.49
	Teleworking	−0.05	0.01	−0.05	−3.99^***^
	Community involvement	0.08	0.02	0.06	4.12^***^
	*R*^2^ = 0.439; *F* = 304.622 (9/3,493), *p* < 0.001				
Model 3—Personal and lifestyle factors	(Constant)	0.69	0.14		4.78^***^
	Age	0.01	0.00	0.13	9.77^***^
	Gender	−0.13	0.03	−0.06	−4.94^***^
	Satisfaction with compensation	0.03	0.03	0.01	0.91
	Ethics and values	0.10	0.02	0.11	6.11^***^
	Psychosocial environment	0.51	0.02	0.52	28.98^***^
	Psychosocial risks related to mental health	−0.02	0.02	−0.02	−1.40
	Physical environment	0.02	0.01	0.02	1.40
	Teleworking	−0.05	0.01	−0.05	−4.07^***^
	Community involvement	0.08	0.02	0.06	4.19^***^
	Risk behaviors	0.01	0.00	0.02	1.38
	Health behaviors	0.03	0.01	0.05	3.44^***^
	Stress management	0.07	0.02	0.05	3.63^***^
	*R*^2^ = 0.443; *F* = 232.140 (12/3,493), *p* < 0.001				

^***^p ≤ 0.001.

Model 2, which focuses on organizational factors, explains 44% of the variance in professional engagement, *F*_(9, 3, 493)_ = 304.622; *p* ≤ 0.001. Being older remains and female remains associated with higher professional engagement. In addition, ethics and values, psychosocial environment, and community involvement also show positive associations, suggesting that organizational contexts that follow ethical principles, support healthy relationships, and engage with the community, promote higher levels of engagement. Conversely, psychosocial risks and telework are negatively associated, indicating that greater psychological demands and physical distance from the workplace tend to reduce professional engagement.

In Model 3, personal and lifestyle factors are included, which explain 44% of the variance in professional engagement, *F*_(12, 3, 493)_ = 232.140; *p* ≤ 0.001. The positive associations with age, ethics and values, psychosocial environment, and community involvement remain significant. Additionally, positive associations are observed between health behaviors and stress management, suggesting that healthier lifestyles and greater stress-regulation capacity are associated with higher levels of professional engagement. Conversely, gender and telework continue to show negative associations.

A differentiated analysis of the four generations under study was then performed: the Baby Boomer generation (Table 4), Generation X (Table 4), Generation Y (Table 5), and Generation Z (Table 6). This analysis aimed to gain a deeper understanding of generational differences in professional engagement.

**Table 4:** Multiple linear regression analysis predicting professional engagement among Baby Boomer professionals.

Models	Variables	*B*	Standard error	Beta	*t*
Model 1—P Sociodemographic factors	(Constant)	−0.23	1.52		−0.15
	Age	0.07	0.02	0.17	2.76^**^
	Gender	−0.24	0.12	−0.12	−2.07^*^
	Satisfaction with compensation	0.26	0.12	0.13	2.18^*^
	*R*^2^ = 0.054; *F* = 6.193 (3/274), *p* < 0.001				
Model 2—P Organizational factors	(Constant)	0.79	1.28		0.62
	Age	0.04	0.02	0.10	2.16^*^
	Gender	−0.15	0.09	−0.07	−1.55
	Satisfaction with compensation	−0.01	0.10	−0.01	−0.12
	Ethics and values	−0.09	0.06	−0.10	−1.37
	Psychosocial environment	0.53	0.07	0.53	7.39^***^
	Psychosocial risks related to mental health	−0.13	0.06	−0.11	−2.13^*^
	Physical environment	0.10	0.05	0.12	2.26^*^
	Teleworking	−0.00	0.05	−0.00	−0.06
	Community involvement	0.18	0.07	0.14	2.78^**^
	*R*^2^ = 0.415; *F* = 22.568 (9/274), *p* < 0.001				
Model 3—P Personal and lifestyle factors	(Constant)	−1.75	1.33		−1.32
	Age	0.05	0.02	0.11	2.33^*^
	Gender	−0.18	0.10	−0.09	−1.93
	Satisfaction with compensation	0.01	0.10	0.00	0.08
	Ethics and values	−0.09	0.06	−0.10	−1.46
	Psychosocial environment	0.52	0.07	0.53	7.34^***^
	Psychosocial risks related to mental health	−0.09	0.06	−0.08	−1.40
	Physical environment	0.10	0.05	0.12	2.29^*^
	Teleworking	−0.03	0.05	−0.03	−0.58
	Community involvement	0.19	0.07	0.15	2.93^**^
	Risk behaviors	0.02	0.01	0.08	1.56
	Health behaviors	0.03	0.03	0.05	0.94
	Stress management	0.12	0.07	0.09	1.80
	*R*^2^ = 0.421; *F* = 17.625 (12/274), *p* < 0.001				

^*^p ≤ 0.05; ^**^p ≤ 0.01; ^***^p ≤ 0.001.

**Table 5:** Multiple linear regression analysis predicting professional engagement among Generarion X.

Models	Variables	*B*	Standard error	Beta	*t*
Model 1—Sociodemographic factors	(Constant)	3.22	0.26		12.39^***^
	Age	0.01	0.01	0.04	1.54
	Gender	−0.09	0.05	−0.05	−2.04^*^
	Satisfaction with compensation	0.48	0.05	0.24	10.54^***^
	*R*^2^ = 0.061; *F* = 39.148 (3/1,765), *p* < 0.001				
Model 2—Organizational factors	(Constant)	1.54	0.25		6.07^***^
	Age	0.01	0.00	0.04	2.39^*^
	Gender	−0.12	0.04	−0.06	−3–29^**^
	Satisfaction with compensation	0.09	0.04	0.05	2.30^*^
	Ethics and values	0.15	0.02	0.16	6.29^***^
	Psychosocial environment	0.44	0.03	0.47	17.64^***^
	Psychosocial risks related to mental health	−0.06	0.02	−0.05	−2.58^**^
	Physical environment	0.03	0.02	0.03	1.40
	Teleworking	−0.05	0.02	−0.05	−2.79^**^
	Community involvement	0.02	0.03	0.02	0.90
	*R*^2^ = 0.399; *F* = 131.195 (9/1,765), *p* < 0.001				
Model 3—Personal and lifestyle factors	(Constant)	1.06	0.28		3.75^***^
	Age	0.01	0.00	0.05	2.54^**^
	Gender	−0.12	0.04	−0.06	−3.24^***^
	Satisfaction with compensation	0.09	0.04	0.04	2.23^*^
	Ethics and values	0.14	0.02	0.16	6.17^***^
	Psychosocial environment	0.43	0.03	0.45	17.06^***^
	Psychosocial risks related to mental health	−0.02	0.02	−0.02	−0.78
	Physical environment	0.02	0.02	0.02	0.99
	Teleworking	−0.05	0.02	−0.05	−2.83^**^
	Community involvement	0.03	0.03	0.02	1.12
	Risk behaviors	0.00	0.01	0.01	0.28
	Health behaviors	0.05	0.01	0.09	4.34^***^
	Stress management	0.03	0.03	0.02	1.26
	*R*^2^ = 0.405; *F* = 101.121 (12/1,765), *p* < 0.001				

^*^p ≤ 0.05; ^**^p ≤ 0.01; ^***^p ≤ 0.001.

**Table 6:** Multiple linear regression analysis predicting professional engagement among Generation Y.

Models	Variables	*B*	Standard error	Beta	*t*
Model 1—Sociodemographic factors	(Constant)	3.10	0.24		12.84^***^
	Age	0.01	0.01	0.05	1.80
	Gender	−0.18	0.06	−0.08	−2.93^**^
	Satisfaction with compensation	0.48	0.06	0.23	8.03^***^
	*R*^2^ = 0.056; *F* = 24.124 (3/1,179), *p* < 0.001				
Model 2—Organizational factors	(Constant)	0.86	0.25		3.45^***^
	Age	0.01	0.01	0.07	3.05^**^
	Gender	−0.13	0.05	−0.06	−2.71^*^
	Satisfaction with compensation	0.03	0.05	0.02	0.64
	Ethics and values	0.09	0.03	0.10	3.24^***^
	Psychosocial environment	0.58	0.03	0.58	19.83^***^
	Psychosocial risks related to mental health	−0.05	0.03	−0.04	−1.75
	Physical environment	−0.01	0.02	−0.01	−0.27
	Teleworking	−0.04	0.02	−0.05	−2.32^*^
	Community involvement	0.11	0.03	0.08	3.48^***^
	*R*^2^ = 0.482; *F* = 122.901 (9/1,179), *p* < 0.001				
Model 3—Personal and lifestyle factors	(Constant)	0.16	0.29		0.53
	Age	0.01	0.01	0.06	2.91^**^
	Gender	−0.15	0.05	−0.07	−3.13^**^
	Satisfaction with compensation	0.02	0.05	0.01	0.37
	Ethics and values	0.09	0.03	0.10	3.25^***^
	Psychosocial environment	0.56	0.03	0.57	19.51^***^
	Psychosocial risks related to mental health	−0.03	0.03	−0.02	−0.91
	Physical environment	−0.00	0.02	−0.00	−0.07
	Teleworking	−0.04	0.02	−0.05	−2.28^*^
	Community involvement	0.11	0.03	0.08	3.63^***^
	Risk behaviors	0.01	0.01	0.05	2.26^*^
	Health behaviors	0.02	0.01	0.03	1.40
	Stress management	0.14	0.03	0.09	4.18^***^
	*R*^2^ = 0.491; *F* = 95.640 (12/1,179), *p* < 0.001				

^*^p ≤ 0.05; ^**^p ≤ 0.01; ^***^p ≤ 0.001.

In Table 3, Model 1 explains 5% of the variance, *F*_(3, 274)_ = 6.193; *p* ≤ 0.001. Age and satisfaction with compensation are each positively associated with engagement: older professionals and those more satisfied with their pay tend to show higher engagement. In contrast, gender is negatively associated with engagement, with men showing lower levels compared to women in this generation.

In Model 2, organizational factors explain 42% of the variance, *F*_(9, 274)_ = 22.568; *p* ≤ 0.001. Engagement among Baby Boomers is positively associated with the psychosocial environment (a healthier work environment and high-quality professional relationships), the physical environment (good working conditions), and community involvement (active connection to the community). In contrast, psychosocial risks are negatively associated with engagement, suggesting that greater psychological or emotional pressure lowers engagement in these professionals.

Model 3, which incorporates demographic, psychosocial, organizational, personal, and lifestyle variables, explains 42% of the variance, *F*_(12, 274)_ = 17.625; *p* ≤ 0.001. Among these, age, the psychosocial environment, the physical environment, and community involvement show positive associations with professional engagement. This suggests that, in this generation, positive work relationships, good physical working conditions, and a strong connection to the community are more strongly linked to professional engagement than personal and lifestyle variables.

In Table 5, the model related to sociodemographic factors (Model 1) explains 6% of the variance in engagement for Generation X, *F*_(3, 1, 765)_ = 39.148; *p* ≤ 0.001. Satisfaction with compensation emerges as a positive predictor, indicating that higher levels of satisfaction contribute to greater engagement among professionals in this generation. Conversely, gender maintains a negative association, with men exhibiting lower levels of engagement.

Model 2, related to organizational factors, explains 40% of the variance. *F*_(9, 1, 765)_ = 131.195; *p* ≤ 0.001. Positive associations are observed with ethics and values and the psychosocial environment, indicating that organizational contexts perceived as ethically conscious and fostering balanced work relationships promote engagement in this generation. In contrast, gender, psychosocial risks, and telework show negative associations, suggesting that these factors are related to lower levels of engagement among Generation X professionals.

Model 3, which incorporates personal and lifestyle factors, explains 41% of the variance, *F*_(12, 1, 765)_ = 101.121; *p* ≤ 0.001. The positive associations with age, ethics and values, and the psychosocial environment are maintained. A notable positive association is observed with health behaviors, suggesting that healthier lifestyle practices are linked to higher levels of professional engagement. Negative associations with gender and telework also persist, reinforcing the impact of these variables in reducing professional engagement in this generation.

In Table 6 the model addressing sociodemographic factors (Model 1) accounts for 6% of the variance in professional engagement, *F*_(3, 1, 179)_ = 24.124; *p* ≤ 0.001. Satisfaction with compensation is positively associated with engagement, underscoring its significance for Generation Y. In contrast, gender is negatively related, indicating men in this generation report lower engagement.

In Model 2, when organizational factors are included, the variance explained increases to 48%, *F*_(9, 1, 179)_ = 122.901; *p* ≤ 0.001. Positive associations are observed with age and the dimensions of ethics and values, psychosocial environment, and community involvement, indicating that within this generation, older professionals, as well as those with favorable perceptions of the ethical climate, work relationships, and community connection, tend to exhibit higher levels of engagement. In contrast, gender and telework show negative associations: being male is associated with lower engagement, and telework (working remotely) is also associated with reduced engagement among Generation Y.

Model 3, which incorporates personal and lifestyle variables, explains 49% of the variance in professional engagement, *F*_(12, 1, 179)_ = 95.640; *p* ≤ 0.001. The positive associations with age, ethics and values, psychosocial environment, and community involvement are maintained. Among personal variables, health behaviors and stress management are positively associated with engagement. Conversely, gender and telework remain negative predictors, reinforcing their influence on engagement in Generation Y.

The sociodemographic factors model (Model 1) presented in Table 7 explains 1% of the variance in professional engagement among Generation Z, *F*_(3, 272)_ = 2.237; *p* ≤ 0.001. Satisfaction with compensation shows a significant positive association, indicating that among younger Generation Z members in the sample, greater pay satisfaction is associated with higher levels of professional engagement.

**Table 7:** Multiple linear regression analysis predicting professional engagement among Generation Z.

Models	Variables	*B*	Standard error	Beta	*t*
Model 1—Sociodemographic factors	(Constant)	2.19	0.85		2.58^**^
	Age	0.05	0.03	0.10	1.55
	Gender	−0.08	0.14	−0.03	−0.54
	Satisfaction with compensation	0.29	0.13	0.13	2.17^*^
	*R*^2^ = 0.013; *F* = 2.237 (3/272), *p* < 0.084				
Model 2—Organizational factors	(Constant)	0.55	0.69		−0.80
	Age	0.05	0.02	0.09	2.01^*^
	Gender	−0.07	0.10	−0.03	−0.71
	Satisfaction with compensation	−0.24	0.11	−0.11	−2.22^*^
	Ethics and values	0.05	0.06	0.05	0.84
	Psychosocial environment	0.73	0.06	0.67	11.55^***^
	Psychosocial risks related to mental health	0.02	0.05	0.02	0.46
	Physical environment	0.00	0.05	0.00	0.07
	Teleworking	−0.08	0.05	−0.08	−1.82
	Community involvement	0.15	0.07	0.11	2.21^*^
	*R*^2^ = 0.484; *F* = 29.393 (9/272), *p* < 0.001				
Model 3—Personal and lifestyle factors	(Constant)	−0.11	0.73		−0.15
	Age	0.05	0.02	0.09	2.02^*^
	Gender	−0.05	0.10	−0.02	−0.44
	Satisfaction with compensation	−0.20	0.11	−0.09	−1.90
	Ethics and values	0.06	0.06	0.06	1.01
	Psychosocial environment	0.75	0.06	0.69	11.59^***^
	Psychosocial risks related to mental health	0.02	0.06	0.02	0.33
	Physical environment	−0.00	0.05	−0.00	−0.04
	Teleworking	−0.08	0.05	−0.08	−1.81
	Community involvement	0.17	0.07	0.12	2.36^*^
	Risk behaviors	−0.02	0.01	−0.06	−1.34
	Health behaviors	−0.05	0.03	−0.08	−1.70
	Stress management	−0.06	0.08	−0.03	−0.75
	*R*^2^ = 0.488; *F* = 22.639 (12/272), *p* < 0.001				

^*^p ≤ 0.05; ^**^p ≤ 0.01; ^***^p ≤ 0.001.

With the inclusion of organizational factors in Model 2, the variance explained increases to 48%, *F*_(9, 272)_ = 29.393; *p* ≤ 0.001. For Generation Z, age, the psychosocial environment, and community involvement are positively associated with engagement, suggesting that the quality of work relationships, social support, and a sense of community contribute to higher engagement. In contrast, satisfaction with compensation shows a negative association, indicating that higher pay satisfaction does not necessarily lead to increased engagement in this group.

Model 3, which incorporates personal and lifestyle factors, explains 49% of the variance, *F*_(12, 272)_ = 22.639; *p* ≤ 0.001. Positive associations with age, psychosocial environment, and community involvement are again observed, reaffirming that, in this generation, positive workplace relationships, community belonging, and increasing age are associated with higher engagement.

## Discussion

By considering individual, organizational, and contextual influences simultaneously, the ecological perspective adopted in this study provides a comprehensive understanding of the factors associated with professional engagement while identifying both shared and generation-specific patterns.

## Sociodemographic factors

A consistent gender difference in professional engagement was observed across the generations analyzed. In several cohorts, women reported slightly higher levels of work engagement than men. This finding is consistent with international evidence (9, 31, 32), suggesting that women are generally more professionally engaged than men under typical working conditions. One possible explanation may lie in functional or social differences. Women in certain roles may demonstrate greater organizational commitment and dedication than men in equivalent positions. Although previous research has highlighted contextual variations, with some countries reporting higher engagement among men and others showing the opposite pattern, the present study identified an overall trend of lower engagement among men across all generations analyzed. These findings suggest that organizations should pay attention to the factors that may contribute to lower engagement among male employees and consider strengthening inclusion and motivation strategies targeted toward this group.

The findings reveal distinct patterns of engagement among Baby Boomers, Generation X, Millennials (Generation Y), and Generation Z regarding professional engagement. Younger workers often report lower levels of engagement with their work and organization than older generations. In contrast, Baby Boomers tend to exhibit relatively high levels of engagement and greater stability in their professional commitment. Millennials and Generation Z are more likely to feel detached from their work, seek new employment opportunities, and place greater value on work that aligns with their personal values. These generational differences highlight the importance of tailoring organizational strategies to the characteristics and expectations of different age groups.

Another finding is that employees who feel fairly rewarded for their work tend to demonstrate greater dedication and organizational commitment. This pattern aligns with evidence showing that a positive evaluation of current remuneration is significantly associated with higher levels of work engagement. Satisfaction with compensation is a key driver of professional engagement across generations. However, the data also reveals generational nuances. Among Baby Boomers, there is a strong positive relationship between satisfaction with compensation and engagement, suggesting that financial reward remains an important motivator for these older workers. For Generation Y, a similar positive association appears, reinforcing the relevance of compensation for engagement. In Generation Z, however, the association is less clear. Satisfaction with compensation contributes to engagement, but this relationship becomes null or negative when organizational and personal factors are considered. One possible interpretation is that younger professionals may prioritize factors such as the work environment and sense of purpose over salary when considering commitment. Nevertheless, fair compensation remains an important workplace resource. Previous research suggests that employees who perceive their pay as fair and competitive tend to report higher levels of engagement, although among younger generations this relationship may depend on the presence of a supportive organizational context (33, 34).

## Organizational factors

Beyond these sociodemographic differences, the findings also underscore the importance of organizational factors in shaping professional engagement. The psychosocial environment and ethics and values, play a significant role in promoting professional engagement, consistent with the JD-R literature. This model aims to analyze how job demands and resources impact employee performance through engagement (35, 36). Across all generations, a healthy work environment rich in relational resources proved to be fundamental in enhancing engagement. Specifically, the quality of the psychosocial environment, characterized by social support, trust, respect, and positive work relationships, was one of the strongest predictors of engagement in all age groups.

This finding aligns with research showing that favorable psychosocial conditions, such as meaningful work, recognition, and a climate of trust, increase engagement and mitigate the negative effects of stress (37, 38). For example, a recent study (39) highlights that work contexts with high psychosocial risks, including conflict, excessive pressure, and toxic relationships, significantly reduce engagement and increase the likelihood of burnout. Conversely, organizations that foster psychological safety and support tend to have more energetic and dedicated employees, resulting in gains in wellbeing and productivity.

In addition to the relational climate, organizational ethics and values emerged as another relevant factor, particularly for the middle generations. In Generation X and Generation Y, the perception of a strong ethical climate, characterized by integrity, fairness, and corporate social responsibility, was positively associated with professional engagement. These results highlight the importance of value congruence and social responsibility for younger and middle-aged workers.

Evidence indicates that a strong ethical climate fosters engagement, with the organization's sense of social responsibility having a particularly pronounced effect on employees' dedication. In contexts where leaders practice ethical leadership and promote transparent, fair policies, employees tend to feel more connected to and committed to their work. Therefore, a positive psychosocial environment and an ethical culture emerge as universal drivers of engagement: employees who feel supported, valued, and aligned with organizational values demonstrate higher levels of vigor, dedication, and absorption in their roles (2, 9).

Mental health emerged as a cross-cutting element in the discussion of professional engagement. Although not always measured directly, it was implicitly reflected through stress and burnout-related variables. Psychosocial risk factors, such as workload, job insecurity, conflicts, or psychological pressure, were found to correlate negatively with engagement, particularly among Baby Boomers and Generation X. These risks affect mental health at work and can lead to emotional exhaustion and cynicism, which undermine employee energy and dedication. This aligns with studies showing that adverse work climates, lacking support and characterized by intense stressors, reduce engagement and increase occupational health problems. Conversely, positive indicators of mental health, such as effective stress management, were associated with higher engagement in younger generations. In Generation Y, for example, individuals reporting effective stress-coping strategies exhibited higher levels of engagement. This suggests that mentally resilient employees with good psycho-emotional balance can maintain greater dedication to their work even in the face of challenges (5, 8, 10, 34).

Additionally, there is evidence that engaged employees tend to have better mental health, suggesting a potentially bidirectional relationship. Psychological wellbeing is both a predictor and an outcome of professional engagement. Work contexts that protect mental health by reducing stressors and providing support promote engagement, while highly engaged employees show lower levels of distress and burnout (5, 39, 40).

## Personal and lifestyle factors

Consistent with the ecological approach, employees' personal health and risk habits were also considered. The results revealed subtle patterns that varied by generation. In Generation X, engaging in positive health behaviors, such as regular physical activity, adequate sleep, and balanced nutrition, was associated with slightly higher professional engagement. Similarly, in Generation Y, engagement was positively correlated with certain moderate-risk behaviors and effective stress management. In this context, “risk behaviors” may include both less healthy habits, such as occasional substance use, and traits related to risk-taking or challenge-seeking. The positive association may reflect that more active, challenge-seeking employees, including those who take calculated risks, also feel more engaged at work. These variables were not significant for Baby Boomers or Generation Z. This may be because older workers have more stable lifestyles, other factors are more influential, or younger workers show limited behavioral variability.

The literature supports links between healthy lifestyles and engagement (41, 42). Engaged employees tend to report healthier habits, such as regular exercise and balanced diets, and are less likely to experience chronic health problems. Conversely, unhealthy lifestyles can undermine energy and focus at work (6, 14, 15). Although the effects are moderate, these findings reinforce that physical wellbeing is closely linked to professional engagement.

Organizations that support employees in developing healthy habits, including physical activity, rest breaks, and wellness programs, promote both health and workplace engagement (42). The role of lifestyle variables in professional engagement can be understood through a personal resources and self-regulation perspective. In the present study, positive health behaviors and stress management were associated with higher professional engagement in the final model, suggesting that engagement is shaped not only by organizational conditions but also by the resources that professionals bring to and maintain within the work context.

Positive health behaviors, such as regular physical activity, adequate sleep, balanced eating patterns, and lower involvement in risk behaviors, may help preserve the energetic, cognitive, and emotional resources required to remain psychologically available and involved at work. Within the Job Demands–Resources framework, these behaviors may be interpreted as personal resources that support vigor, persistence, concentration, and emotional regulation when professionals face demanding work conditions. Recent evidence supports this interpretation. Kiema-Junes et al. (43) found that higher levels of physical activity were associated with greater work engagement, whereas sedentary behavior was linked to lower engagement and reduced vigor and dedication. These findings suggest that lifestyle behaviors may contribute to engagement by enhancing energy, vitality, and recovery capacity.

Similarly, Sonnentag et al. (44) conceptualized the relationship between work and health behaviors as bidirectional, demonstrating that work can either facilitate or hinder physical activity and healthy eating, while these behaviors may, in turn, influence workers' functioning and wellbeing. This perspective is particularly relevant for the present study, as it supports an ecological interpretation of lifestyle: health behaviors are not merely individual choices, but are shaped by working conditions, time availability, workload, organizational culture, and opportunities for recovery. Stress management may operate through a complementary psychological mechanism. Professionals who are better able to regulate stress are more likely to sustain attention, emotional balance, constructive coping, and a sense of control in demanding work contexts. These processes may help explain the positive association between stress management and professional engagement. However, this finding should not be interpreted as placing responsibility for engagement solely on individual workers. Consistent with a public health and healthy workplace perspective, lifestyle promotion and stress management are most effective when supported by organizational environments that minimize preventable psychosocial risks, facilitate recovery, promote autonomy, and enable healthy choices. Accordingly, lifestyle behaviors should be understood as part of the broader work–health interface, in which personal resources and organizational conditions interact to sustain professional engagement.

## Closer look at generational differences

Across all four generations, a healthy psychosocial work environment and organizational support were associated with higher engagement, whereas psychosocial risks and ethical misalignment undermined it. Satisfaction with compensation also contributed to engagement in most generations, although its relative importance varied, consistent with previous literature (39).

Additionally, the importance of being part of a work context with good physical conditions and strong community ties was highlighted. This suggests that the most engaged Baby Boomers perceived not only a supportive internal environment but also an active organizational connection to the wider community, such as participation in social initiatives, reflecting the values of this generation regarding contribution and belonging. Generation X placed particular importance on organizational ethics, while work–life balance also emerged as a key factor, reflected in the positive association with health behaviors and the negative impact of excessive telework on engagement. This suggests that members of Generation X may experience isolation or lack of structure in remote work and prefer in-person interaction to feel fully engaged.

Generation Y (Millennials) showed a multidimensional profile. Similar to Generation X, they valued a strong ethical organizational climate and positive work relationships, while engagement was also associated with effective stress management and opportunities for challenge. Engaged Millennials tend to seek meaning in their work and opportunities for growth. It is therefore unsurprising that their engagement is higher when they feel aligned with organizational values and can simultaneously develop personal resilience skills.

Generation Z showed a somewhat different pattern. Although social support remained important across generations, support and integration within the work group were particularly relevant for this cohort. Satisfaction with compensation lost explanatory power for their engagement when considered alongside other factors, suggesting that Generation Z prioritizes a positive work environment, support, and a sense of purpose, potentially over remuneration. Telework, and lifestyle variables were not significantly associated with engagement among Generation Z, possibly reflecting greater familiarity with remote work and the cohort's lower heterogeneity or shorter exposure to professional habits.

In summary, all generations benefit from positive organizational resources and are negatively affected by psychosocial risks, although the relative importance of specific factors varies. Older generations tend to prioritize stability and financial recognition, whereas younger generations place greater emphasis on ethics, personal development, work–life balance, and alignment with social purpose. These generational patterns partially support commonly reported stereotypes, such as Millennials seeking meaningful work and frequent feedback and Baby Boomers demonstrating loyalty when they feel valued. However, these differences are not absolute, as the fundamental drivers of work engagement (e.g., respect, safety, and a sense of achievement) are shared across all generations. It is important to note that these findings should also be interpreted with caution when considering the distinction between generational, age, and career-stage effects. In the present study, generational groups were defined according to birth-year cohorts, which means that observed differences may partly reflect age, professional experience, or career-stage characteristics rather than purely generational attributes. This is a well-recognized limitation in cross-sectional generational research, as age, period, and cohort effects are difficult to disentangle when data are collected at a single point in time. Recent evidence also warns against deterministic interpretations of generational categories, showing that many assumed generational differences in work-related attitudes are small, inconsistent, or may reflect broader age-related and contextual processes rather than stable cohort traits (45).

Nevertheless, the present findings may still be meaningfully interpreted through a cautious generational lens. The different patterns observed among Generation Y and Generation Z, particularly the stronger relevance of psychosocial climate, community involvement, purpose, and support, are consistent with recent longitudinal evidence suggesting that some work-related expectations among younger cohorts may reflect cohort-based socialization rather than only temporary career-stage effects. For example, Egerová et al. (46) found that differences in work-related expectations between Generation Y and Generation Z were mainly influenced by cohort effects and remained relatively stable during the turbulent period from 2019 to 2022. Therefore, in the present study, generational categories should not be understood as fixed or homogeneous psychological profiles, but as useful analytical markers of workers who entered employment under different social, technological, and organizational conditions.

Accordingly, the results should be interpreted as reflecting generation-related patterns, rather than definitive generational effects. Older workers' stronger association with stability, fair compensation, physical conditions, and community connection may reflect both cohort socialization and accumulated career experience. Younger workers' stronger sensitivity to psychosocial climate, ethical values, social support, flexibility, and purpose may reflect both their early career stage and broader cohort experiences shaped by digitalization, changing employment expectations, and increased attention to mental health and work–life balance. Future longitudinal and age–period–cohort studies are needed to clarify whether these differences remain stable over time or evolve as workers progress through their careers.

## Theoretical and International Implications

Although the present study was conducted in Portugal, its contribution extends beyond the national context. The challenges examined are not country-specific but reflect broader transformations in contemporary labor markets. Across the world, organizations are increasingly facing issues related to mental health at work, psychosocial risks, demographic aging, digitalization, hybrid and remote work, labor mobility, and the coexistence of multiple generations in the workplace. International organizations have recognized mental health at work as a public health and labor policy priority, emphasizing the shared responsibility of governments, employers, workers, and civil society to prevent psychosocial risks and promote healthier work environments (47). Similarly, the OECD (48) has highlighted the need for labor market policies and organizational practices that promote employability, job quality, lifelong learning, age-inclusive workplaces, and the participation of diverse workforce groups, including young people, older workers, women, and migrants.

From this perspective, Portugal provides a relevant empirical setting for examining processes that are occurring internationally. Like many other European and OECD countries, Portugal is experiencing changes in work organization, the expansion of digital and hybrid forms of work, increased labor mobility, and growing pressure on organizations to retain workers while protecting mental health and wellbeing. Accordingly, the findings contribute to the international work engagement literature by showing that professional engagement is not only an individual motivational state but also an ecological outcome shaped by organizational resources, psychosocial conditions, ethical climate, work arrangements, health-related resources, and generation-related expectations.

This study also extends existing theoretical models of employee engagement by integrating three dimensions that are often examined separately: organizational context, individual health-related resources, and generational patterns. In line with the Job Demands–Resources model and healthy workplace frameworks, the results suggest that engagement is strengthened when organizations provide ethical, supportive, and psychologically safe environments and is weakened when workers are exposed to psychosocial risks or poorly structured work arrangements. Moreover, the generational analysis adds nuance to existing engagement models by suggesting that the relative importance of these resources may vary according to age, career stage, and cohort-related work expectations.

Finally, the study contributes to the international literature by linking research, organizational diagnosis, and policy-oriented action. Embedded in the Portuguese Laboratory of Healthy Workplaces (LABPATS), the study is part of a broader applied research agenda focused on assessing, monitoring, and improving healthy workplaces. This practice-oriented approach has international relevance because it translates theoretical models of engagement and occupational health into actionable evidence for organizations and policymakers. Accordingly, the Portuguese case may inform other countries and institutions seeking to develop evidence-based, multilevel strategies to promote professional engagement, mental health, and sustainable work environments.

## Implications for organizations and practical recommendations

The findings of this study suggest that professional engagement should be understood not only as an individual motivational outcome but also as an indicator of the quality and sustainability of work environments. Across generations, engagement was consistently associated with modifiable organizational conditions, particularly the psychosocial environment, organizational ethics and values, community involvement, fair compensation, work arrangements, and, in some groups, health behaviors and stress management. These findings have several practical implications for organizations and policymakers.

For organizational practitioners, the first recommendation is to assess engagement and psychosocial risks regularly using validated tools and a continuous improvement approach. Assessment should not be limited to measuring engagement as an outcome but should also identify the organizational conditions that support or undermine it, including workload, role clarity, leadership practices, interpersonal relationships, psychological safety, perceived fairness, and ethical climate. The results should be discussed with managers and workers and translated into concrete action plans with clearly defined priorities, responsibilities, timelines, and follow-up indicators. These action plans should include interventions aimed at strengthening the psychosocial climate, such as promoting supportive leadership, open communication, performance recognition, and team-building initiatives, which can foster a climate of trust and cooperation that enhances engagement across generations.

Second, organizations should move beyond isolated wellbeing initiatives and adopt a work-design approach. Activities such as stress management sessions, mindfulness, or wellness programs may be beneficial, but they are insufficient if excessive job demands, poor communication, interpersonal conflict, low autonomy, or lack of recognition remain unaddressed. Practical interventions should therefore include reviewing workloads, strengthening role clarity, improving communication channels, preventing harassment and interpersonal conflict, and ensuring access to supportive supervision. This approach is particularly important because psychosocial risks were negatively associated with engagement. Providing access to psychological counseling or mindfulness training can be particularly beneficial for supporting Millennials and Generation Z, who experience higher levels of anxiety and value employers who are attentive to work-life balance. Similarly, simple initiatives, such as encouraging active breaks, offering flexible exercise schedules, or promoting internal health challenges, can increase healthy behaviors and, consequently, energy and dedication at work.

Third, leadership development should be central to engagement strategies. Managers should be trained in healthy and ethical leadership, constructive feedback, recognition, conflict management, and the early identification of psychological distress. Regular check-ins, participatory decision-making, and visible recognition of workers' contributions can strengthen trust, belonging, and organizational commitment. These practices are relevant across generations, although younger workers may be particularly sensitive to the quality of relationships, support, and alignment with organizational values.

Fourth, hybrid and telework policies should be designed carefully. The negative association between telework and engagement in some generational groups suggests that remote work should not be viewed as either inherently beneficial or harmful. Organizations should develop structured hybrid models that preserve flexibility while maintaining opportunities for social connection, mentoring, informal learning, and team cohesion. For Generation X and Generation Y, regular in-person collaboration may help reduce feelings of isolation and disconnection, while establishing regular in-office days may further strengthen social interaction, mentoring, and knowledge sharing. For Generation Z, effective onboarding, mentoring, and opportunities to connect with the organization's purpose are particularly important, especially in digital or hybrid work contexts. Although organizations can leverage this generation's technological fluency by offering flexible work arrangements, these practices should be complemented by structured integration initiatives to foster engagement in remote work environments.

Fifth, engagement strategies should be generation-sensitive without becoming stereotyped. Older workers may place greater value on stability, fair compensation, physical working conditions, and recognition of their experience, whereas younger workers may be more responsive to the psychosocial climate, ethical values, growth opportunities, and a sense of purpose. Organizations can address these differences through flexible career development, intergenerational mentoring, reverse mentoring, inclusive recognition practices, and opportunities to participate in organizational decision-making. Although fair remuneration alone is unlikely to guarantee engagement, particularly among younger generations, transparent salary review mechanisms, appropriate benefits, and non-financial rewards, such as recognition and development opportunities, can strengthen motivation by addressing both extrinsic and intrinsic needs.

For policymakers, these findings reinforce the importance of treating healthy work environments as a public health priority. Policies should support the systematic assessment and prevention of psychosocial risks, the promotion of mental health, and professional engagement across sectors.

National or sectoral monitoring systems could help identify vulnerable groups and guide evidence-informed interventions, particularly when data are analyzed by age group, gender, work modality, sector, and health status. Policymakers should also provide technical and financial support for organizations, especially small and medium-sized enterprises, to implement healthy workplace programs. This support may include access to validated assessment tools, training for managers, implementation guidelines, certification or recognition mechanisms, and support for pilot interventions that can later be scaled up. Clearer guidance on hybrid work is also needed, including recommendations on the right to disconnect, workload management, ergonomic conditions, the inclusion of remote workers, and opportunities for social and professional integration.

Finally, occupational health policies should adopt a life-course and intergenerational perspective. Supporting younger workers' transition into employment, promoting lifelong learning, retaining older workers, and adapting work for professionals with chronic health conditions are all relevant to sustaining engagement over time. Overall, the study supports an ecological approach in which engagement is promoted through coordinated action at multiple levels: individual resources, team relationships, leadership practices, organizational culture, working conditions, and public policy.

In summary, organizations that balance fair extrinsic rewards with strong investment in psychosocial resources and wellbeing are better positioned to engage employees across generations. This ecological approach, addressing the individual (health, skills), the team (support, relationships), and organizational culture (values, policies), promotes higher engagement while reducing burnout and turnover, contributing to more sustainable and productive work environments (9, 29, 49).

## Limitations

The findings reinforce and extend existing theories of work engagement by incorporating a generational and multilevel perspective. They confirm the importance of psychosocial and organizational factors highlighted in models such as the Job Demands–Resources (JD-R) model, while showing that their relative influence may vary across age groups and cohorts. These results suggest that conceptual models of engagement could benefit from considering moderating variables such as generation or career stage, supporting the view that work engagement evolves throughout the professional life course rather than remaining static. Furthermore, the ecological perspective adopted in this study highlights that multiple levels, from individual to societal, interact to shape engagement.

Despite these contributions, some limitations should be acknowledged. The present study has some limitations that should be acknowledged. First, the cross-sectional nature of the data captures associations at a single point in time. Therefore, it is not possible to infer direct causal relationships. For example, it cannot be stated with certainty whether engaging in healthy behaviors leads to higher engagement or whether more engaged professionals tend to take better care of their health. Future longitudinal studies would be valuable to clarify directionality and the evolution of engagement across the life course. Second, the measures relied on participant self-reports, which carry a risk of response bias. For instance, social desirability may have led to underreporting of risk behaviors, particularly among older groups. It may also have caused over reporting of self-declared engagement. Triangulation with objective data, such as absenteeism or performance metrics, could further strengthen the conclusions.

Moreover, the exclusive reliance on self-reported measures may have increased the risk of common method variance, as both predictors and outcomes were collected using the same method. Although this approach is common in large-scale occupational and public health research, future studies could further strengthen the robustness of findings by integrating multi-source or objective indicators and by adopting longitudinal designs. Additionally, the operationalization of “generations” was based on approximate age ranges. While useful for analysis, these categories may obscure intra-generational differences and do not capture the full heterogeneity within each group (for example, not all Millennials share the same attitudes). Cohort definitions and uneven sample sizes, such as the overrepresentation of Generation X compared to Generation Z, may have influenced the detection of effects. Future studies should aim for balanced samples and consider both age and cohort effects.

A further limitation relates to the study's geographic and cultural focus: it examined professionals in the Portuguese context, which may have distinct labor-market characteristics and organizational cultures. The findings and recommendations should be interpreted within this context, and generalizations to other countries should be made with caution, although many results align with international literature.

It is also important to acknowledge that the analytical strategy has certain limitations. Although hierarchical multiple regression was appropriate for the exploratory aims of the present study and allowed us to estimate the relative contribution of sociodemographic, organizational, and personal/lifestyle factors across generations, this approach does not test a full latent structural model. Given that several variables reflect psychological and organizational constructs, future research should examine more specific theoretical models using Structural Equation Modeling. SEM would allow researchers to test direct and indirect pathways, account for measurement error, and explore mediation and moderation mechanisms linking psychosocial risks, organizational resources, health-related variables, and professional engagement. Longitudinal designs would be particularly valuable to clarify the directionality of these associations.

## Conclusion

From a broader perspective, this study makes several contributions to the literature on professional engagement. First, it adopts an ecological approach that integrates organizational, psychosocial, and health-related factors within a single analytical framework. Second, it provides evidence from a large and diverse sample of professionals, allowing for a robust examination of engagement across multiple generations. Third, by exploring generational differences, the study adds nuance to existing engagement models, showing that while some drivers are shared across cohorts, their relative importance varies according to generational context and career stage. Thus, this study provides a comprehensive analysis of professional engagement from a multigenerational and ecological perspective, identifying both common patterns and generation-specific differences among Baby Boomers, Generation X, Generation Y, and Generation Z.

The findings show that work engagement is shaped not only by job design or individual characteristics but also by a broader ecological system that includes lifestyle habits, social support, organizational policies, and the psychosocial work environment. This reinforces the connection between organizational behavior, occupational health, and community wellbeing, while supporting the value of interdisciplinary approaches to understanding engagement across generations.

From a practical perspective, these findings provide valuable insights for managers, human resources professionals, occupational health and safety specialists, and other organizational stakeholders. Improving work engagement requires coordinated actions across multiple domains. In people management, this includes developing leaders' intergenerational competencies, fostering a supportive organizational climate, recognizing employees' contributions, and promoting meaningful work. In occupational health management, it involves implementing programs tailored to the physical and mental health needs of different age groups, ranging from burnout prevention for younger workers to ergonomic interventions for older employees. Reward management should balance fair compensation with flexibility, development opportunities, and other non-monetary benefits increasingly valued by younger generations. Organizations that fail to address these differences risk losing younger employees, who tend to be less tolerant of poor work environments, while underutilizing the experience of older workers, who can serve as mentors and cultural anchors. In contrast, organizations that implement targeted strategies are more likely to foster engaged, loyal, and productive teams while reducing turnover, presenteeism, and workplace conflict (50–52).

The findings also highlight the importance of integrating psychosocial factors into occupational health and safety policies and interventions, as addressing physical risks alone is insufficient to promote engagement, wellbeing, and safety. More broadly, organizations seeking sustainable and healthy workplaces should align their policies and practices with the needs of a multigenerational workforce through interventions that promote individual wellbeing, supportive leadership, effective work design, and inclusive, socially responsible organizational practices. An integrated approach sharpens engagement, boosts wellbeing, reduces generational conflict, and draws out the full potential of every age group, supporting a harmonious and productive workplace. Adopting an ecological perspective that considers individual, interpersonal, organizational, community, and policy influences can strengthen engagement, enhance wellbeing, reduce generational conflict, and maximize the potential of employees across all age groups.

## Data Availability

The raw data supporting the conclusions of this article will be made available by the authors, without undue reservation.

## References

[1] Anttila A Nuutinen M Van Gils M Pekki A Sauni R. Associations of depressive symptoms and psychosocial working conditions with sickness absences in a Finnish cohort of 11,495 employees. Prev Med Rep. (2024) 47:102899. doi: 10.1016/j.pmedr.2024.102899 39444590 PMC11497368

[2] Backhaus I Lohmann-Haislah A Burr H Nielsen K di Tecco C Dragano N. Organizational change: challenges for workplace psychosocial risks and employee mental health. BMC Public Health. (2024) 24:2477. doi: 10.1186/s12889-024-19815-w 39261822 PMC11389294

[3] Mátó V Tarkó K Lippai L Nagymajtényi L Paulik E. Psychosocial work environment risk factors among university employees–a cross-sectional study in Hungary. Zdr Varst. (2020) 60:10. doi: 10.2478/sjph-2021-0003 33488817 PMC7780769

[4] Vykopalova H. Prevalence of psychosocial risk severity, stress and cognitive dissonance in working conditions. J Loss Prev Process Ind. (2025) 94:105552. doi: 10.1016/j.jlp.2025.105552

[5] Gaspar T Guedes FB Cerqueira A Baban A Rus C Matos MG. Burnout as a multidimensional phenomenon: how can workplaces be healthy environments? J. Public Health. (2024) 33:2591–604. doi: 10.1007/s10389-024-02223-0

[6] Assander S Bergström A Olt H Guidetti S Boström AM. Individual and organisational factors in the psychosocial work environment are associated with home care staffs' job strain: a Swedish cross-sectional study. BMC Health Serv Res. (2022) 22:1418. doi: 10.1186/s12913-022-08699-4 36434716 PMC9701045

[7] Björk-Fant JM Bolander P Forsman AK. Work–life balance and work engagement across the European workforce: a comparative analysis of welfare states. Eur J Public Health. (2023) 33:430–4. doi: 10.1093/eurpub/ckad046 36952280 PMC10234642

[8] Pinto A Carvalho C Mónico LS Moio I Alves J Lima TM. Assessing psychosocial work conditions: preliminary validation of the Portuguese short version of the Copenhagen psychosocial questionnaire III. Sustainability. (2024) 16:7479. doi: 10.3390/su16177479

[9] Gaspar T Salado V Machado M Guedes FB Correia M Gaspar MG. The healthy workplaces ecosystems and professionals' stress management during the COVID-19 pandemic. Sustainability. (2023) 15:11432. doi: 10.3390/su151411432

[10] Whitsed C Girardi A Fitzgerald S Williams J. Exploring academic staff engagement in a time of crisis and change through the lens of a multilevel job demand-resources analysis. J High Educ Policy Manag. (2024) 47:1–19. doi: 10.1080/1360080X.2024.2371076

[11] Cortes ML Louzado JA Oliveira MG Bezerra VM Mistro S Medeiros et al. Association between perceived stress and health-risk behaviors in workers. Psychol. Health Med. (2022) 27:746–60. doi: 10.1080/13548506.2020.1859567 33295792

[12] Smithdorf G Reynders J Meyer E November R Malema M. The assessment of health risk behaviors among the administrative staff at an institution of higher education. Open Public Health J. (2022) 15:1–7. doi: 10.2174/18749445-v15-e2208100

[13] Bourke M Wang HFW McNaughton SA Thomas G Firth J Trott M . Clusters of healthy lifestyle behaviors are associated with symptoms of depression, anxiety, and psychological distress: a systematic review and meta-analysis of observational studies. Clin Psychol Rev. (2025) 118:102585. doi: 10.1016/j.cpr.2025.102585 40239241

[14] Jackson AT Mazzola JJ Loveless JP Gargurevich R Jang H Cheon SH. The impact of healthy choices at work: daily barriers, facilitators, and their effects on stress and performance. Occup Health Sci. (2025) 9:383–406. doi: 10.1007/s41542-025-00220-7

[15] Sarwar F Panatik SA Sukor MSM Rusbadrol N. A job demand–resource model of satisfaction with work–family balance among academic faculty: mediating roles of psychological capital, work-to-family conflict, and enrichment. Sage Open. (2021) 11:21582440211006142. doi: 10.1177/21582440211006142

[16] Mudrak J Zabrodska K Kveton P Jelinek M Blatny M Solcova I . Occupational well-being among university faculty: a job demands-resources model. Res High Educ. (2018) 59:325–48. doi: 10.1007/s11162-017-9467-x

[17] Dilekçi Ü Limon I Manap A Alkhulayfi AMA Yildirim M. The association between teachers' positive instructional emotions and job performance: work engagement as a mediator. Acta Psychol. (2025) 254:104880. doi: 10.1016/j.actpsy.2025.104880 40069988

[18] Schaufeli WB. Work engagement in Europe: relations with national economy, governance and culture. Organ Dyn. (2018) 47:99–106. doi: 10.1016/j.orgdyn.2018.01.003

[19] Schaufeli W. Engaging leadership: how to promote work engagement? Front Psychol. (2021) 12:754556. doi: 10.3389/fpsyg.2021.754556 34777155 PMC8578900

[20] Schaufeli WB Salanova M González-Romá V Bakker AB. The measurement of engagement and burnout: a two sample confirmatory factor analytic approach. J Happiness Stud. (2002) 3:71–92. doi: 10.1023/A:1015630930326

[21] Chakradhar K Waddill PJ Kleinhans KA. Resilience and the multigenerational academic work environment in the United States. J Intergener Relat. (2018) 16:374–94. doi: 10.1080/15350770.2018.1489332

[22] Andrade MS Westover JH. Generational differences in work quality characteristics and job satisfaction. In: Evidence-Based HRM: A Global Forum for Empirical Scholarship, vol. 6. Emerald Publishing Limited (2018). p. 287–304. doi: 10.1108/EBHRM-03-2018-0020

[23] Culp-Roche A Hampton D Hensley A Wilson J Thaxton-Wiggins A Otts JA . Generational differences in faculty and student comfort with technology use. SAGE Open Nurs. (2020) 6:2377960820941394. doi: 10.1177/2377960820941394 33415296 PMC7774396

[24] Huyler D Gomez L Rocco TS Plakhotnik MS. Leading different generational cohorts in the workplace: focus on situational and inclusive leadership. New Horiz Adult Educ Hum Res Dev. (2025) 37:6–19. doi: 10.1177/19394225241297230

[25] Silva J Carvalho A. The work values of Portuguese generation z in the higher education-to-work transition phase. Soc Sci. (2021) 10:297. doi: 10.3390/socsci10080297

[26] Durrani N Makhmetova Z. A multi-layered socio-ecological framework for investigating teacher well-being: key predictors and protective factors. Sustainability. (2025) 17:900. doi: 10.3390/su17030900

[27] Gaspar T Telo E Jesus S Xavier M Dias MC Matos MC . Laboratório Português de Ambientes de Trabalho Saudável: Evolução da Saúde Ocupacional 2021 a 2024 [Portuguese Laboratory of Healthy Work Environments: Evolution of Worker Health 2021 to 2024] (eBook). Laboratório Português de Ambientes de Trabalho Saudáveis (LABPATS) (2025). Available online at: https://53237d59-ec33-49aa-bc7f-86826566ffa8.filesusr.com/ugd/645903_f5066c993e2a459ea9f73858a856d (Accessed February, 10, 2026).

[28] Gaspar T Faia-Correia M Machado MC Xavier M Guedes FB Pais-Ribeiro J . Ecossistemas dos ambientes de trabalho saudáveis (EATS): instrumento de avaliação dos healthy workplaces. Psicol Saúde Doenças. (2022) 23:252–68. doi: 10.15309/22psd230124

[29] World Health Organization. Healthy Workplaces: A Model for Action for Employers, Workers, Policy-Makers and Practitioners (2010). Available online at: https://iris.who.int/server/api/core/bitstreams/a803f0ee-559e-4625-9611-e71ac3e8a9ba/content (Accessed February, 21, 2026).

[30] Otufowora AA Striley CW Vaddiparti K Scicchitano MJ Cottler LB. Study navigation and enrollment in a community sample. J Intergener Relatsh. (2022) 21:299–320. doi: 10.1080/15350770.2022.2041526 37724159 PMC10506841

[31] Islam MN Furuoka F Idris A. Influence of gender diversity on employee work engagement in the context of organizational change: evidence from Bangladeshi employees. Int J Asian Bus Inf Manag. (2021) 12:1–19. doi: 10.4018/IJABIM.294099

[32] OECD. Gender Equality at Work – Gender Equality in a Changing World: Taking Stock and Moving Forward (2025). Available online at: https://www.oecd.org/content/dam/oecd/en/publications/reports/2025/05/gender-equality-in-a-changing-world_5a0af5ef/e808086f-en.pdf (Accessed February, 17, 2026).

[33] Kulikowski K Sedlak P. Can you buy work engagement? The relationship between pay, fringe benefits, financial bonuses and work engagement. Curr Psychol. (2020) 39:343–53. doi: 10.1007/s12144-017-9768-4

[34] Malik MHA Musah AA. The role of intergenerational differences in influencing perceptions of salary and benefits: a review study. J Glob Econ Bus Finance. (2024) 6:1–10. doi: 10.53469/jgebf.2024.06(09)0.01

[35] Bakker AB Demerouti E Sanz-Vergel A. Job demands–resources theory: ten years later. Annu Rev Organ Psychol Organ Behav. (2023) 10:25–53. doi: 10.1146/annurev-orgpsych-120920-053933

[36] Demerouti E Bakker AB. Job demands-resources theory in times of crises: new propositions. Organ Psychol Rev. (2023) 13:209–36. doi: 10.1177/20413866221135022

[37] Inoue A Eguchi H Kachi Y Tsutsumi A. Perceived psychosocial safety climate, psychological distress, and work engagement in Japanese employees: a cross-sectional mediation analysis of job demands and job resources. J Occup Health. (2023) 65:e12405. doi: 10.1002/1348-9585.12405 37218064 PMC10203353

[38] Tsoni E Lazanaki V Katsaros K. The influence of organizational climate on work engagement: evidence from the Greek industrial sector. Adm Sci. (2025) 15:413. doi: 10.3390/admsci15110413

[39] Lara-Moreno R Ogallar-Blanco AI Guzmán-Raya N Vázquez-Pérez ML. The exhaustion triangle: how psychosocial risks, engagement, and burnout impact workplace well-being. Behav Sci. (2025) 15:408. doi: 10.3390/bs15040408 40282031 PMC12024121

[40] Jindo T Kai Y Kitano N Tsunoda K Nagamatsu T Arao T. Relationship of workplace exercise with work engagement and psychological distress in employees: a cross-sectional study from the MYLS study. Prev Med Rep. (2020) 17:101030. doi: 10.1016/j.pmedr.2019.101030 31890476 PMC6931185

[41] Cortés-Denia D Lopez-Zafra E Pulido-Martos M. Physical and psychological health relations to engagement and vigor at work: a PRISMA-compliant systematic review. Curr Psychol. (2023) 42:765–80. doi: 10.1007/s12144-021-01450-y

[42] Matsuoka J Nagata T Odagami K Adi NP Nagata M Kajiki S . Number of good lifestyle habits affects higher work engagement over the next year: a retrospective cohort study. J Occup Environ Med. (2025) 67:911–5. doi: 10.1097/JOM.0000000000003467 40490358

[43] Kiema-Junes H Saarinen A Korpelainen R Kangas M Ala-Mursula L Pyky R Hintsanen M. More physical activity, more work engagement? A Northern Finland Birth Cohort 1966 study. J Occup Environ Med. (2022) 64:541–9. doi: 10.1097/JOM.0000000000002530 35260539 PMC9301987

[44] Sonnentag S Kottwitz MU Koch TJS Völker J. Enrichment and conflict between work and health behaviors: new scales for assessing how work relates to physical exercise and healthy eating. OSH. (2023) 7:251–96. doi: 10.1007/s41542-022-00134-8

[45] Ravid DM Costanza DP Romero MR. Generational differences at work? A meta-analysis and qualitative investigation. J Organ Behav. (2024) 46:43–65. doi: 10.1002/job.2827

[46] Egerová D Komárková L Rotenbornová L. Generational differences in work-related expectations: examining period and cohort effects. Econ Sociol. (2024) 17:103–17. doi: 10.14254/2071-789X.2024/17-4/6

[47] World Health Organization and International Labour Organization. Mental Health at Work: Policy Brief . World Health Organization (2022). Available online at: https://www.who.int/publications/i/item/9789240057944 (Accessed July 10, 2026).

[48] OECD. OECD Employment Outlook 2025: Can We Get Through the Demographic Crunch? Paris: OECD Publishing (2025).

[49] World Health Organization. Mental Health at Work: Policy Brief . World Health Organization (2022). Available online at: https://www.who.int/publications/i/item/9789240057944 (Accessed February, 21, 2026).

[50] Costa N Oliveira CM Ferreira P. How to measure the happy-productive worker thesis. In: People Management - Highlighting Futures (2022). p. 1–14. doi: 10.5772/intechopen.107429

[51] Costa N Ferreira P Miguel Oliveira C. A bibliometric approach to the thesis of the happy-productive worker—a journey through the concepts and measurement. Sage Open. (2024) 14:1–16. doi: 10.1177/21582440241249549

[52] Salas-Vallina A Pozo-Hidalgo M Gil-Monte PR. Are happy workers more productive? The mediating role of service-skill use. Front Psychol. (2020) 11:456. doi: 10.3389/fpsyg.2020.00456 32292366 PMC7120033

[53] Gaspar T Telo E Arriaga M Sousa B Jesus S Xavier MC . Relatório Ambientes de Trabalho Saudáveis: Estudo das diferenças de género e geração [Healthy Work Environments Report: Study of Gender and Generation Diferences]. Laboratório Português de Ambientes de Trabalho Saudáveis (LABPATS) (2024). Available online at: https://53237d59-ec33-49aa-bc7f-86826566ffa8.filesusr.com/ugd/645903_f7eba44e45bb45319642858ebf5b28a4.pdf (Accessed February, 10, 2026).

[54] Cohen S Kamarck T Mermelstein R. A global measure of perceived stress. J Health Soc Behav. (1983) 24:385–96. doi: 10.2307/2136404 6668417

[55] Bronfenbrenner U. Towards an experimental ecology of human development. Am Psychol. (1977) 32:513–31. doi: 10.1037/0003-066X.32.7.513

[56] Bronfenbrenner U. The Ecology of Human Development. Harvard University Press (1979).

[57] Bronfenbrenner U Morris PA. The Bioecological Model of Human Development. In:Lerner RM Damon W, editors. Handbook of child psychology: Theoretical models of human development. Hoboken, NJ: John Wiley & Sons Inc (2006) 793–828.

[58] Cantril H. The pattern of human concerns. New Jersey: Rutgers University Press (1965).

[59] Limone P Toto GA. Factors that predispose undergraduates to mental issues: A cumulative literature review for future research perspectives. Front. Public health (2022) 10:831349. doi: 10.3389/fpubh.2022.831349 35252101 PMC8888451

[60] Rosa EM Tudge J. Urie Bronfenbrenner's theory of human development: Its evolution from ecology to bioecology. J Fam Theory Rev. (2013) 5:243–58. doi: 10.1111/jftr.12022

